# Predicting stress response and improved protein overproduction in *Bacillus subtilis*

**DOI:** 10.1038/s41540-022-00259-0

**Published:** 2022-12-27

**Authors:** Juan D. Tibocha-Bonilla, Cristal Zuñiga, Asama Lekbua, Colton Lloyd, Kevin Rychel, Katie Short, Karsten Zengler

**Affiliations:** 1 Bioinformatics and Systems Biology Graduate Program, University of California, San Diego, 9500 Gilman Drive, La Jolla, CA 92093-0760 USA; 2 Department of Pediatrics, University of California, San Diego, 9500 Gilman Drive, La Jolla, CA 92093-0760 USA; 3 Division of Biological Sciences, University of California, San Diego, La Jolla, CA USA; 4 Department of Bioengineering, University of California, San Diego, La Jolla, CA 92093-0412 USA; 5 Center for Microbiome Innovation, University of California, San Diego, 9500 Gilman Drive, La Jolla, CA 92093-0403 USA

**Keywords:** Metabolic engineering, Biochemical networks, Computer modelling

## Abstract

*Bacillus subtilis* is a well-characterized microorganism and a model for the study of Gram-positive bacteria. The bacterium can produce proteins at high densities and yields, which has made it valuable for industrial bioproduction. Like other cell factories, metabolic modeling of *B. subtilis* has discovered ways to optimize its metabolism toward various applications. The first genome-scale metabolic model (M-model) of *B. subtilis* was published more than a decade ago and has been applied extensively to understand metabolism, to predict growth phenotypes, and served as a template to reconstruct models for other Gram-positive bacteria. However, M-models are ill-suited to simulate the production and secretion of proteins as well as their proteomic response to stress. Thus, a new generation of metabolic models, known as metabolism and gene expression models (ME-models), has been initiated. Here, we describe the reconstruction and validation of a ME model of *B. subtilis*, *i*JT964-ME. This model achieved higher performance scores on the prediction of gene essentiality as compared to the M-model. We successfully validated the model by integrating physiological and omics data associated with gene expression responses to ethanol and salt stress. The model further identified the mechanism by which tryptophan synthesis is upregulated under ethanol stress. Further, we employed *i*JT964-ME to predict amylase production rates under two different growth conditions. We analyzed these flux distributions and identified key metabolic pathways that permitted the increase in amylase production. Models like *i*JT964-ME enable the study of proteomic response to stress and the illustrate the potential for optimizing protein production in bacteria.

## Introduction

*Bacillus subtilis* is the best-studied Gram-positive bacterium and has promising industrial applications^1^. The organism has been widely used in industrial applications, including for the production of antibiotics, enzymes, and vitamins^2^. Furthermore, *B. subtilis* serves as a model in studies of gut^3^ and soil microbiome^4^, for our understanding of sporulation and cell differentiation, biofilm formation, as well as to unravel pathogenicity in related pathogens^5,6^.

The tremendous amount of omics and physiological data available for *B. subtilis* allowed reconstructing one of the first bacterial genome-scale metabolic models (M-models)^7^. The M-model consists of a network of all known metabolic reactions, resulting in high prediction accuracy of gene essentiality, growth on different carbon and nitrogen substrates, and gene-knockout phenotypes^8^. Even though this network can accurately predict metabolic responses to nutrient levels and gene knockouts^9,10^, enzyme production costs, and protein secretion are out of the scope of the M-model. Moreover, the biomass composition is a constraint in M-models, thus limiting predictions about variations of biomass precursor abundances^11^. Therefore, it is impracticable to simulate stress conditions that involve shifts in gene expression or alterations in biomass composition with the M-model. While M-model coupling with metabolomics data has been successfully employed to analyze variations in biomass composition^12,13^, no a priori prediction of these shifts has been possible with bacterial M-models.

A new generation of computational models enables linking gene expression mechanisms to metabolic reactions^14^. The models of metabolism and gene expression (ME-models) link enzyme production profiles with metabolic reaction fluxes, thus assigning additional protein biosynthetic costs to metabolism. Now, predicted metabolic fluxes account for optimal proteome composition at specific growth conditions. ME-models can also be associated with the chaperones framework to simulate changes in the proteome in response to temperature or metal availability^15–18^. The first ME model was reconstructed for *Thermotoga maritima*^18^, with explicit definitions of necessary coupling constraints for complex usage, transcription, translation, and mRNA degradation. The next ME-models were reconstructed for *Escherichia coli*, the first being published by Thiele et al.^19^, which then underwent three subsequent updates, namely *i*OL1650-ME^14^, *i*JL1678-ME^17^, and *i*JL1678b-ME^20^. The last of these was released with a new standard on ME-model reconstruction, called COBRAme, upon which this work was based.

Here, we reconstructed the ME model of *B. subtilis* str. 168, *i*JT964-ME, based on the available M-model *i*YO844^7^, gene annotation in BsubCyc^21^, and extensive manual curation of transporters and secretory pathways. We show the increased predictive capability of *i*JT964-ME to simulate gene essentiality, stress-induced biomass composition variation, and shifts in gene expression. Furthermore, we deployed the ME model to accurately predict enzyme production under various conditions, showcasing its ability to assist protein production strategies.

## Results

### Properties and benchmarking of the metabolic and gene expression model of *B. subtilis*, *i*JT964-ME

**Fig. 1:**
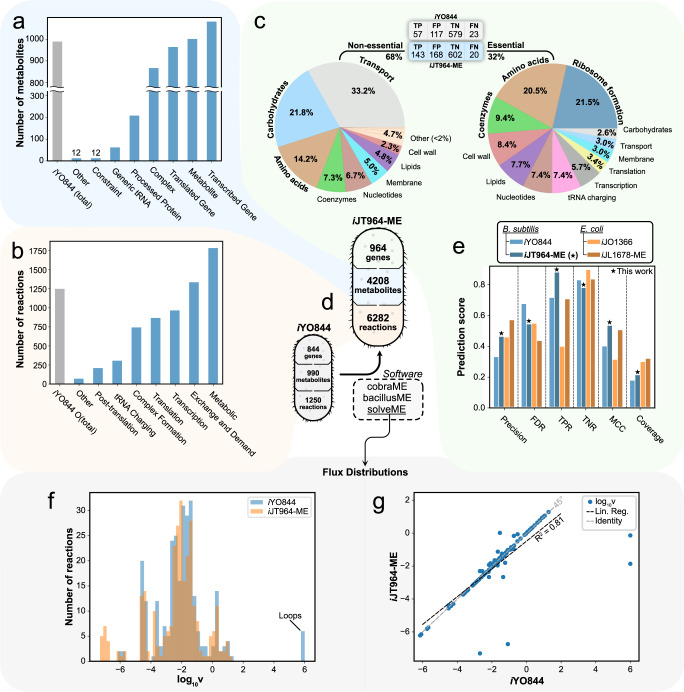
Properties of *i*JT964-ME and gene essentiality prediction performance. **a** Breakdown of metabolite types included in the model. Complex: active enzyme, Constraint: biomass component, Generic tRNA: tRNA, Metabolite: standard metabolite, Processed Protein: monomer before complexation, Transcribed Gene: mRNA, Translated Gene: protein before any modification. **b** Breakdown of reaction types included in the model. Metabolic: reactions inherited from *i*YO844, Exchange and Demand: inlet and outlets of the model, Transcription: mRNA synthesis, Translation: protein synthesis, Complex Formation: complex synthesis, tRNA charging: charged tRNA synthesis, Post-translation: protein modification, Other: biomass constraints. **c** ME-model predictions of gene essentiality; distribution of essential and non-essential genes. In between the pie charts, a table shows the confusion matrix for the essentiality predictions in *i*YO844 and *i*JT964-ME. **d** Number of genes, metabolites, and reactions included in the model. **e** Accuracy scores for predictions of gene essentiality by *B. subtilis* and *E. coli* models (M- and ME-models). Predictions were contrasted with the reported essential genes by Juhas et al.^41^ for *B. subtilis* and in EcoCyc^42^ for *E. coli*. Score calculations of Precision, False Discovery Rate (FDR), True Positive Rate (TPR), True Negative Rate (TNR), and Matthews Correlation Coefficient (MCC) are explained in “Methods”. **f** Histograms showing the flux distributions of metabolic reactions for both *i*YO844 and *i*JT964-ME. Considering the vast range in orders of magnitude across fluxes, here we show their distribution in terms of ${{{\mathrm{log}}}}_{{{{\mathrm{10}}}}}{{{\mathrm{v}}}}$, with ***v*** representing flux (see “Methods”). **g** Comparison in terms of ${{{\mathrm{log}}}}_{{{{\mathrm{10}}}}}{{{\mathrm{v}}}}$ of the metabolic reactions in *i*YO844 and *i*JT964-ME. Correlation is shown with a linear regression and a Pearson correlation coefficient (*R*^2^).

Reconstruction of *i*JT964-ME was performed by adapting the available metabolic modeling packages COBRAme^20^, COBRApy^22^, and ECOLIme^20^. COBRAme functions were altered to be compatible with *Bacillus subtilis* gene and protein nomenclature used in GenBank, FASTA, and other files (Table 1). The resulting pipeline expanded the existing *B. subtilis* M-model *i*YO844 with non-metabolic reactions, including translation, transcription, tRNA charging, and post-translational modification^20^. The final ME model (*i*JT964-ME) contains 964 genes, 6282 reactions, and 4208 metabolites (Fig. 1d). A detailed breakdown of metabolite and reaction types is shown in Fig. 1a, b. The ME-model components that are inherited from the M-model’s metabolic network are called “metabolic”, such as the “metabolic reaction” and “metabolite” in Fig. 1a, b. In this study, we used *i*YO844 as a template M-model, with modifications following updated information on transport reactions and gene-protein-reaction associations. A complete list of updates is provided in Supplementary Table 1. Approximately 28% of the network in *i*JT964-ME comprises metabolic reactions, with 23% of metabolites resulting from these metabolic reactions. Note that the number of metabolic reactions in *i*JT964-ME is far greater (5023 additional reactions) than in *i*YO844, since reversible reactions are split into forward and reverse subreactions.

**Table 1:** Information included in the *B. subtilis* ME-model *i*JT964-ME.

Information type	Description/Notes	Containing script	Source
Core metabolic network	Stoichiometric matrix, metabolic reactions, and metabolites included in the network. Exchange reaction constraints are adopted as well.	*generate_flatfiles*	*i*YO844^7^
Gene-reaction rules	Enzyme-reaction associations were taken from the available M-model *i*YO844. Several new transporters were added and corrected as listed in Supplementary Table 1.	*Metacyc_dependent_files*	*i*YO844^7^
Genome	Genbank file containing gene names, positions, compositions, lengths, and primary structure.	*build_me_model*	Genbank
Protein complexes	All possible protein complexes in *B. subtilis*, as well as which monomers that are contained in each complex.	*Metacyc_dependent_files*	BsubCyc^23^
Protein stoichiometry	Monomer composition of complexes. All available enzyme stoichiometries in BsubCyc were used, while unavailable ones were defined by homology with *E. coli*.	*Metacyc_dependent_files*	BsubCyc^23^ EcoCyc^24^
Post-translational modification	Protein modification information was taken from BsubCyc and was also defined by homology with *E. coli*.	*Metacyc_dependent_files*	BsubCyc^23^ EcoCyc^24^
Transcription Units	List of transcription unit names, lengths, positions, strands, sigma factors, and rho dependence.	*Metacyc_dependent_files*	BsubCyc^23^
Cleaved methionine	List of proteins that undergo N-terminal methionine excision.	*Metacyc_dependent_files*	BsubCyc^23^
Ribosome composition and synthesis	Subunit composition of the ribosome and synthesis subreactions.	*build_me_model*	SubtiWiki^25^ BsubCyc^23^
Protein compartment and secretory pathway	Final compartment of translated proteins and the secretory pathways by which they reach their final destinations. Tat-pathway signal peptides were predicted by SignalP 5.0.	*Metacyc_dependent_files*	BsubCyc^23^ SignalP 5.0^26^
rRNA modifications	List of rRNA modifications and catalyzing enzymes.	*ribosome*	Desmolaize et al.^27^
RNA degradosome	Composition of the RNA degradosome	*transcription*	Lehnik-Habrink et al.^28^
Excision machinery	Composition of rRNA-containing, monocistronic, and polycistronic rRNA machinery.	*transcription*	BsubCyc^23^
Initiation, elongation, and termination subreactions	Translation initiation, elongation, and termination subreactions.	*translation*	BsubCyc^23^
Codon usage	Codon usage table for *B. subtilis*.	*build_me_model*	Nakamura et al.^29^
Protein folding	Independent and GroEL-dependent folding.	*translation*	Endo & Kurusu^30^
Translocation pathways	Mechanism of translocation: Sec-SRP, SRP, and Tat pathways. Sec-SRP, SRP, and Tat translocation pathways were described by Simonen & Palva^31^ (Sec-SRP and SRP) and Fu et al.^32^ (Tat).	*translation*	Fu et al.^32^ Simonen & Palva^31^
Enzyme turnover rates (K_eff_)	Coefficients that link enzyme usage with reaction fluxes, as described by Lloyd et al.^20^. Effective coefficients ($K_{eff}$) were assigned depending on the enzyme’s role in primary or secondary metabolism, as reported by Bar-Even et al.^33^.	*build_me_model*	Bar-Even et al.^33^

*i*JT964-ME includes new transcription and translation reactions, which correspond to 28% of the total reactions. An additional 23% of reactions represent complex formation (including generic complexes), post-translational modification, and tRNA charging, and the remaining reactions (21%) account for exchange and demand reactions. While most exchange reactions are kept the same as in *i*YO844, 1,081 new demand reactions were added to account for RNA degradation. To test the quality of the ME-metabolic network, we ran a high-throughput phenotypic analysis of 87 carbon sources based on experimental results from ref. ^7^, which were initially used to validate *i*YO844. *i*JT964-ME and *i*YO844 simulations achieved a Matthews Correlation Coefficient (MCC) of 0.454. Considering the base metabolic network of the model is fully inherited from *i*YO844, it is expected that growth calls on different substrates varies little to none. Major changes, however, are to be expected in predictions at the level of gene expression and protein synthesis. Complete lists of flux distributions for the M- and ME- models are provided in Supplementary Data 1.

Coupling transcription and translation rates to metabolic fluxes allows the ME model to deal with artificially high fluxes and metabolic loops. In M-models, artificial loops are inevitably predicted as a way for the model to maximize the metabolite transport and energy production. By linking protein biosynthesis pathways to fluxes, ME-models penalize unrealistic fluxes and can predict biologically relevant alternatives. An example of this is depicted in Fig. 1f. A handful of reactions showed artificially high fluxes in the M-model (~10^6^), which is not the case for *i*JT964-ME. On the other hand, the rest of the predicted metabolic fluxes are distributed similarly across the network in both *i*YO844, with each reaction carrying a comparable flux in both models, yielding an overall Pearson correlation coefficient (*R*^2^) of 0.81 (Fig. 1g).

The addition of gene expression reactions into the network of *B. subtilis* resulted in a 14% increase in genome coverage (total 964 genes out of 4443 coding genes), with 32% of them being essential in the growth on glucose as predicted by *i*JT964-ME (Fig. 1e). The extensive manual curation performed for *i*JT964-ME significantly increased the prediction scores of gene essentiality. In some cases, prediction scores surpass those predicted for the *E. coli* M- and ME-models. Interestingly, just a 14% increase in gene content allowed *i*JT964-ME to predict essentiality with increases of 34% in the Matthews Correlation Coefficient (MCC) and 40% in Precision. The superior performance achieved by ME-models can be explained by the emergent metabolite-metabolite and metabolite-protein dependencies that arise when flux in the network is permitted to alter gene expression profiles.

Figure 1c shows that genes associated with transport reactions compose the most considerable portion of non-essential genes. *B. subtilis* is a versatile organism that can metabolize a wide range of different carbon and nitrogen sources. This results in the model containing many transporters, though most of them are not active under specific growth conditions. For example, if glucose is supplemented in minimal medium the transporter for glucose-6-phosphate will be prioritized. The next significant groups of non-essential genes correspond to carbohydrate and amino acid metabolism. Even though both biomass precursors are essential for growth, *B. subtilis* contains several alternative pathways to synthesize them. This is especially the case for carbohydrate metabolism, as only 6% of its genes were predicted to be essential and relate mostly to the pentose phosphate pathway (PPP) and glycolysis reactions. In a similar way, alternative pathways render some reactions in nucleotide metabolism, lipid metabolism, membrane synthesis, and cell wall synthesis non-essential.

As biomass precursor synthesis reactions were split into essential and non-essential, cofactors associated with those reactions fell into both categories accordingly (“Coenzymes” in Fig. 1c). Almost a third of genes encoding for cofactor metabolism were found to be essential. Furthermore, a significant portion of essential genes (21%) is related to amino acid synthesis for protein synthesis and growth (“Amino acids” in Fig. 1c). Notably, the entirety of the gene expression machinery was predicted to be essential, which includes ribosome formation (21.5%), transcription (5.7%), translation (3.4%), and tRNA charging (7.4%). It is worthy to note that essentiality is overestimated in ME-models for gene expression machinery, as all complexes in this category are formed by a fixed number of subunits that must be complete to have a nonzero production flux and ultimately growth. For example, *rpsT* was observed to not be essential^23^; however, since it is annotated as a subunit of the ribosome, it is essential to carry out translation in the ME-model. As a result of this limitation, false positives are higher among the core expression machinery (Supplementary Fig. 1) and the TNR scores decrease by 6.0 and 7.5% in *i*JT964-ME and *i*JL1678-ME, respectively (Fig. 1e).

### Predicting upregulation of tryptophan synthesis under ethanol stress

Stress by increased concentrations of fermentation products, e.g., alcohols or short-chain fatty acids, is one of the principal stresses to overcome in industrial settings^24^. A machine learning algorithm was recently applied to identify groups of genes in *B. subtilis* with significant differential expression in experimental transcriptomics datasets across several stress conditions^25^. The results highlighted a group of significantly co-regulated genes associated with an 8–20% upregulation of tryptophan biosynthetic genes under ethanol stress (4% v/v). The *trp* operon (trpEDCFBA-hisC-tyrA-aroE) codes for enzymes that carry out tryptophan biosynthesis. It has been suggested that *trp* upregulation is caused by ethanol inducing a decrease in tryptophan concentrations^25^, although it has not been fully explained yet. We performed simulations to predict ethanol stress and give a mechanistic insight into gene expression phenomena in *B. subtilis*.

**Fig. 2:**
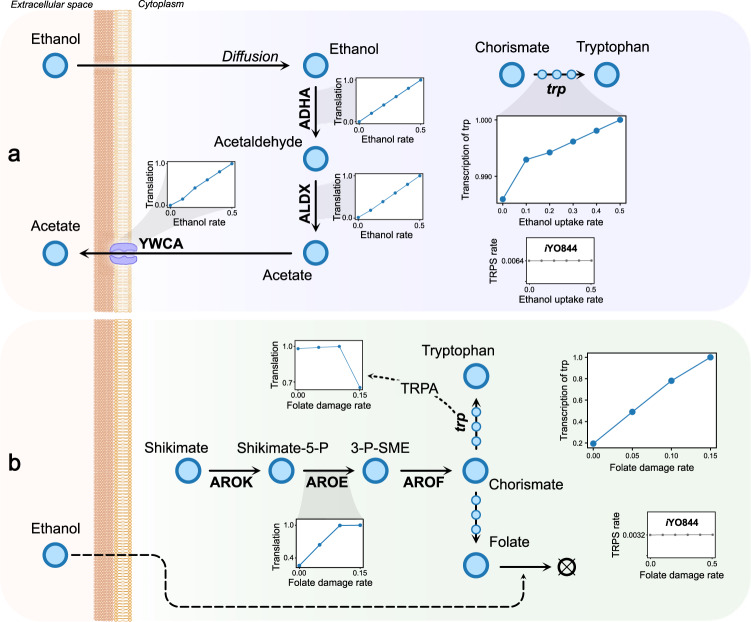
Upregulation of tryptophan synthesis under ethanol stress. **a** Hypothesis for the cause of upregulation as predicted by simulations. Results show that ethanol triggers an increased amino acid demand for the synthesis of enzymes necessary for ethanol breakdown, such as adhA and aldX, and the acetate exporter ycwA. The M-model (*i*YO844) predictions, the separate graph in gray, show no change in tryptophan synthesis. **b** Previously reported hypothesis by Rychel et al. as tested by our model. Our simulations suggest that the *trp* gene aroE has an increased expression to replenish damaged folate, which increments *trp* transcription. All translation and transcription rates are shown as fractions of the maximum value obtained in the observed range. Ethanol and folate damage rates have units of mmol gDW^−1^ h^−1^. Tryptophan synthesis results as predicted by *i*YO844 are shown in a separate graph in gray.

*i*JT964-ME contains transport reactions that simulate the flow of ethanol through the cell membrane via diffusion as experimentally observed. We simulated ethanol diffusion rates between (0 and 0.5 mmol/gDW/h). In agreement with the recent differential expression results^25^, the model predicted the increase in tryptophan synthesis due to increased ethanol uptake (Fig. 2a). In our simulations, transcription of *trp* genes was predicted to increase due to ethanol uptake, although translation rates varied, as shown in detail in Fig. 2b. While trpE and trpF translation was downregulated (Supplementary Fig. 2), expression of the proteins trpD, trpC, trpB, and trpA was upregulated to increase fluxes through anthranilate phosphoribosyltransferase (trpD), indole-3-glycerol-phosphate synthase (trpC), and tryptophan synthase (trpBA). Similarly, aroE and tyrA expression increased as a response to a higher demand for shikimate dehydrogenase (aroE) and prephenate dehydrogenase (tyrA). When we used the same constraints in the M-model *i*YO844 predictions yielded no variation in tryptophan synthesis (Fig. 2a).

Our simulations show that the higher tryptophan demand can be caused solely by an increase in the demand for ethanol processing and acetate secretion enzymes (Fig. 2a). According to in silico experiments, ethanol was converted to acetate through alcohol dehydrogenase (adhA) and aldehyde dehydrogenase (aldX), which was then secreted through a sodium-dependent acetate symporter (ywcA). The translation of ywcA caused ~65% of the total increment in tryptophan synthesis rate, 6.5% by adhA, and aldX. In comparison, the remaining 28.5% was distributed almost evenly across gene and protein expression machinery, e.g., RNA polymerase and ribosomes.

Under the hypothesis that tryptophan concentration driving *trp* expression^25^, the flux of its precursor chorismate would be redirected to synthesize folate, which is consumed by ethanol oxidation byproducts. This mechanism has not yet been well-described^26^. However, model simulations of folate depletion showed a significant increase in *trp* transcription (Fig. 2b). Transcription of *trp* was predicted to increase due to higher demand of aroE for chorismate synthesis, despite tryptophan synthesis decreasing.

### *i*JT964-ME reproduces regulation of gene expression under salt stress

**Fig. 3:**
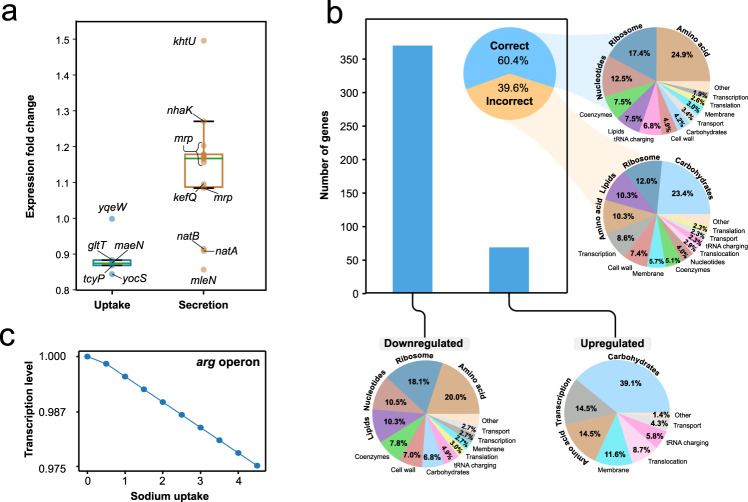
Prediction of differential expression under salt stress. **a** Fold change in transcription of sodium exporters and importers, as reported in the RNASeq dataset available^45^. **b** Accuracy of differential expression prediction under salt stress. Breakdowns of metabolic functions of differentially regulated reactions are shown in the pie charts. **c** Relative transcription level of the *arg* operon under increasing sodium uptake rates, from 0 to 4 ${{{\mathrm{mmol}}}}\,{{{\mathrm{gDW}}}}^{ - 1}\,{{{\mathrm{h}}}}^{ - 1}$.

The ability to overcome osmotic stress defines how competitive an organism is in under high salinity. Salt stress in *B. subtilis* is an ongoing research area, with the primary objective of understanding how cells are affected by excess ions and how they adapt to it. Both biophysical and metabolic responses have been identified^27^ with repercussions at the industrial scale^28^. We deployed *i*JT964-ME to unravel metabolic mechanisms to overcome osmotic stress. We used transcriptomics data of *B. subtilis* growing under salt stress^29^ to evaluate the predicted flux distributions at the genome scale. We modeled salt stress with excess sodium uptake (see “Methods”). Sodium is moved through the membrane via active transport, so transport fluxes in the model trigger transporter expression. However, sodium importers are downregulated (Fig. 3a), while sodium exporters are upregulated to help pump out the excess ion and maintain internal homeostasis (Fig. 3a). Therefore, to model salt stress, we forced an excess influx of sodium with no coupled enzymatic expression, while secretion was left coupled to the synthesis of its transporter.

Figure 3b shows the differential expression of active genes as predicted by *i*JT964-ME and reported in *SubtiWiki*. *i*JT964-ME accurately captured the regulation of 60.4% of differentially expressed genes. The model accurately captures the response of most genes associated with the main metabolic pathways, such as amino acid synthesis, ribosome formation, and nucleotide synthesis. These pathways are accurately captured since their activity is related to the organism’s core metabolic response to stress. However, some genes of core metabolism were incorrectly predicted, though they correspond to a minority within their respective subsystems. For example, 79% of amino acid synthetic genes and 70% of ribosome formation genes were accurately captured. On the other hand, most incorrectly predicted genes correspond to secondary metabolism, such as carbohydrate metabolism, cell wall synthesis, and the transcription of the genes involved in those reactions. More specifically, 74 and 33% (Supplementary Fig. 3) of downregulated and upregulated genes were predicted correctly, respectively. The decrease in accuracy in upregulated genes is largely caused by incorrectly predicted carbohydrate-related genes (Fig. 3b). In M- and ME-models, the prediction of differential expression of storage compounds, e.g., carbohydrates and lipids, is particularly complicated, as storage is largely linked to sub-optimal growth and transition to dormancy^30^. Under stress conditions, storage compound biosynthetic pathways are directed by complex regulatory signals, which are currently out of the capabilities of a metabolic model.

Our predictions of differential expression agree with the recent observations in transcriptomics^25^, which described an unexpected and previously unexplained downregulation of arginine synthesis under salt stress conditions. As shown in Fig. 3c, our model accurately predicts the decrease in expression of the *arg* operon when excess sodium enters the cell. This reduction is explained by the model as a part of a general downregulation of all amino acid synthesis, resulting from a salt-induced decreased capacity of protein synthesis.

### Optimization of protein secretion

*B. subtilis*, classified as Generally Recognized as Safe (GRAS) by the FDA^5^, is a widely used cell factory for the production of proteins due to its accelerated metabolism and highly efficient secretory pathways. One of the most prominent biotechnological application of *B. subtilis* is the production of amylase for a variety of materials and detergents industries^31^. Amylase production and secretion in this organism has been extensively optimized, mainly through random mutagenesis and variation in starch-feeding strategies^31,32^.

M-models have successfully been used to identify critical mutations and growth medium compositions to optimize the production of metabolism of interest, e.g., lipids in microalgae^33^. While M-models can accurately capture the biosynthetic mechanism of metabolic compounds, optimization of enzyme synthesis is not feasible by these models. ME-models on the other hand have great potential for the simulation and optimization of protein synthesis and secretion. In this work, we manually curated the translocation pathways of *B. subtilis* (Table 1). Thereupon, we illustrate the ability of the ME model *i*JT964-ME to predict and optimize the production and secretion of amylase.

**Fig. 4:**
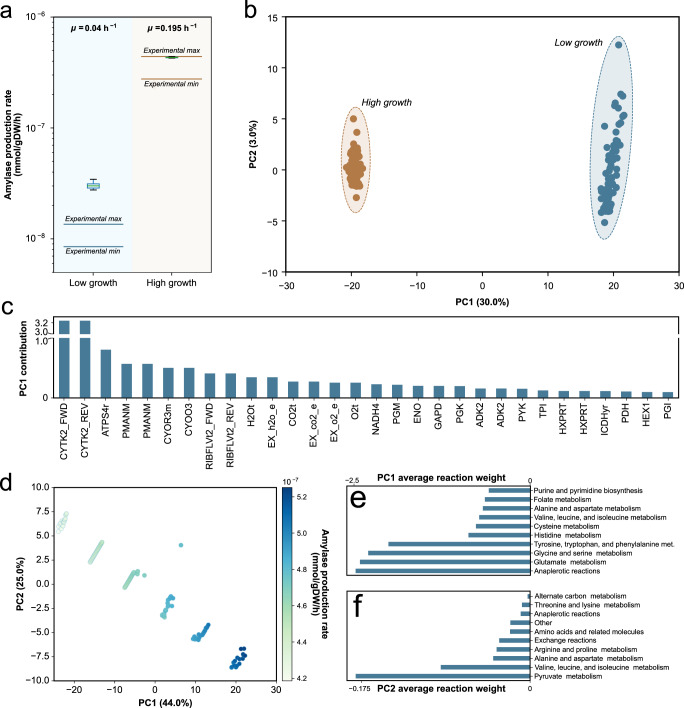
Prediction of amylase secretion. **a** Prediction of amylase secretion rate at two different growth rates. The distribution of amylase production rates was calculated by sampling the solution space close to the optimal growth rate (see “Methods”). Low growth data was taken from ref. ^31^, at a growth rate of 0.04 h^−1^. High growth data was taken from ref. ^32^, at a growth rate of 0.195 h^−1^. The molar amylase production rate was calculated from reported activity data (see “Methods”) and presented in log scale on the *y* axis, **b** PCA plot of the sampling with the first two components in low and high growth conditions. **c** Average reaction contributions to the difference of PC1 position of samples. **d** Sampling of the overexpression of amylase at high growth (see “Methods”). The samples are color-coded with their respective amylase secretion rate with units of ${{{\mathrm{mmol}}}}\,{{{\mathrm{gDW}}}}^{ - 1}{{{\mathrm{h}}}}^{ - 1}$. **e**, **f** The bar plots show the average reaction weight of subsystems in the two principal components, PC1 (**e**) and PC2 (**f**), that describe the highest variance in the sampling of amylase overexpression.

First, we collected two available datasets describing the time-course biomass and amylase concentrations at low (0.04 h^−1^)^31^ and high (0.195 h^−1^) growth rate^32^. Simulations were then performed with starch as the carbon source, which triggers the secretion of amyE in the model. Starch uptake rates were set to fit the experimental growth rate described in the mentioned kinetic studies. Then, we sampled the solution space close to the optimal solution (above 90% the optimal growth rate) to generate a distribution representing biologically relevant secretion rates robustly^12,34^ (see “Methods”). The predicted secretion rates at both conditions were compared with the experimental data (Fig. 4a).

Under low growth, the model overestimates the amylase secretion rate, while *i*JT964-ME predicts secretion rates within the reported experimental ranges at high growth rate. The discrepancy at the low growth rate condition is not surprising, as metabolic models inherently cannot capture several regulatory processes under sub-optimal growth^35^. Nonetheless, the model can predict a steep increment in amylase secretion rates necessary to sustain a higher growth rate. To illustrate the biological insight that *i*JT964-ME can provide, we performed a Principal Component Analysis (PCA) on the simulated data. As expected, both growth conditions cluster tidily in the PCA plot shown in Fig. 4b. The largest principal component (PC1) that was identified explained 30% of the variance, with the most significant contributions coming from nucleotide, organic carbon breakdown, and energy metabolism reactions, e.g., cytidilate kinase (CYTK2), phosphomannomutase (PNANM), ubiquinol-cytochrome oxidoreductase (CYOR), and ATP synthase (ATPS4r) (Fig. 4c). Since both conditions occur at two very different metabolic activities, it is expected that the difference in the fluxes of biomass precursor synthesis, organic carbon assimilation, and energy production describes the most considerable portion of the variance. While the reactions with significant contribution to PC1 could be targeted with mutations for the optimization of *B. subtilis* growth and amylase secretion in starch, we further designed an in silico experiment to isolate the effect of amyE expression at a constant growth rate.

**Fig. 5:**
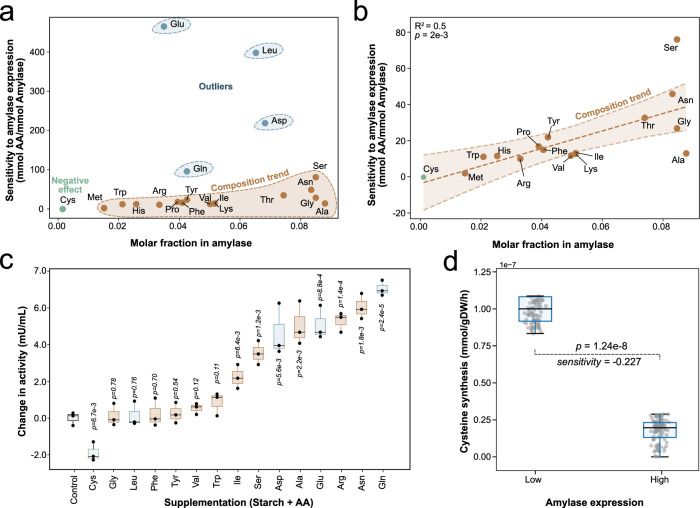
Validation of amylase overexpression. **a** Correlation between molar fraction of amino acids in amylase structure with their predicted sensitivity in amylase overexpression. Sensitivity was calculated as the relative change in amino acid synthesis because of a forced increase in amylase secretion, with units of mmol amino acid per mmol of secreted amylase. **b** Correlation between molar fraction of amino acids with their sensitivity without predicted outliers with a 95% confidence interval. **c** Change in amylase activity after supplementation of amino acids (see “Methods”). Activity was measured with a colorimetric assay at 405 nm, and then the average value of starch-only was subtracted from all samples to obtain the true effect of supplementation. All samples included starch and an amino acid (except for the control, with only starch and no amino acid). Significance in the change of amylase production compared to the control was assessed with a two-tailed *t* test (*P* values are shown on top of the box plots). **d** Sampling of cysteine synthesis rates at low and high-amylase secretion conditions (see “Methods”). Significance in the change of cysteine synthesis rates in low and high-amylase expression conditions was assessed with a two-tailed *t* test (*P* value shown). The sensitivity of cysteine synthesis to amylase expression is calculated as the ratio of change between these rates (see “Methods”).

We tested what groups of reactions would significantly drive the overexpression of amyE by fixing the growth rate at the lowest of the high growth conditions and forcing amyE overproduction until reaching the highest secretion rate. With this new sampling dataset, we performed a PCA that generated a profound insight into the effect of amyE overexpression on the network. The two largest components (PC1 and PC2 in Fig. 4d) described a strikingly higher portion of the variance (69%). As expected, both components mostly consist of amino acid synthesis reactions (Fig. 4d). PC1 is described by valine, leucine, isoleucine, alanine, aspartate, arginine, and proline. On the other hand, PC2 consists of glutamate, glycine, serine, tyrosine, tryptophan, and phenylalanine. Interestingly, these 11 amino acids do not correspond to those with the highest composition in the amylase primary structure (Fig. 5a). For example, the most influential amino acid in PC1 is glutamate, though its composition is less than half (3.5%) than alanine (8.8%). A similar trend is observed in PC2, where the highest-ranking amino acids are valine, leucine, and isoleucine, which lie only among the top 10 (5%, 6.5%, 5.15%, respectively). This indicates that, although their composition in the protein is not nearly as high as alanine, their biosynthetic pathways might pose a bottleneck to target in the overexpression and industrial production of amylase.

Figure 5a shows the individual effect of amylase overexpression on the synthesis of amino acids and shows its correlation with the molar fraction in the protein structure. Glutamine, glutamate, leucine, and aspartate stand out as outliers from this expected trend. Discarding these outliers, the molar fraction showed a significant correlation with predicted sensitivity (*R*^2^ = 0.5, *P* = 0.02). A few more amino acids are called in Fig. 5b as outliers, such as serine and alanine, although their deviation is orders of magnitude less than the previous outliers.

We aimed to test the effect of outliers in Fig. 5a that were predicted to influence amylase overexpression in a way that does not follow the expected trend linked to molar fraction in amylase (Fig. 5b). *B. subtilis* was grown in M9 medium supplemented with starch and different amino acids (see “Methods”), and the resulting amylase expression was quantified using enzymatic activity as a proxy for protein content (see “Methods”), as described in the previous studies^36^. A complete summary of sensitivity, composition, and experimental values is provided in Supplementary Data 2. Raw measurements of amylase activity (OD_405_) and biomass concentration (OD_600_) are provided in Supplementary Data 3 and Supplementary Data 5, respectively.

Predicted low-sensitivity amino acids, such as tryptophan, phenylalanine, tyrosine, valine, and isoleucine, were also observed to induce a significantly lower increase in amylase activity. Interestingly, the model predicted that cysteine would have a negative effect of −0.23 (mmol Cys) (mmol Amylase)^−1^, it being the only amino acid with a negative predicted effect. The observed amylase activity (Fig. 5c) with added cysteine was lower than the control with only starch, which confirms the prediction of this amino acid being the only one that decreases amylase activity. We performed sampling at high- and low- amylase secretion rates and assessed the cysteine synthesis flux to ensure that the negative signal of our predictions was robust. Flux sampling results at low and high-amylase secretion are presented in Supplementary Data 4. Figure 5d shows that the cysteine synthesis is significantly negatively correlated with amylase secretion (*P* = 1.24e-8), with a mean ratio of −0.227 (mmol Cys) (mmol Amylase)^−1^, which is in sync with the single optimum shown in Fig. 5a.

On the other hand, high-sensitivity amino acids with a medium-level composition, such as glutamate and aspartate, were shown to have some of the highest and most statistically significant effects on amylase activity. Moreover, the colorimetric assay confirmed that glutamine would show a remarkable effect despite having an average abundance in the composition of amylase. In some cases, supplementation effect agrees with growth rate changes, as some high-sensitivity amino acids induced a significantly higher growth rate and vice versa (Supplementary Fig. 4). Therefore, the effect could sometimes be explained by the nutritional value of the amino acids to *B. subtilis*, in terms of energetic level and macronutrient content. It can also be argued that higher amylase secretion rates favor the uptake of starch, and thus, biomass production. However, this is not the case for the negative effect of cysteine, which indicates it is not related with its nutritional value. Further, aspartate, glutamine and glutamate, showed an amylase secretion effect comparable to asparagine (Fig. 5c) but their growth rate effects were significantly lower (Supplementary Fig. 4). These results show that *i*JT964-ME can capture effects of supplementation on protein secretion beyond the individual effects of the composition trend (Fig. 5b), as well as the nutritional value of the supplementation.

Inevitably, discrepancies were encountered between model simulations and observed amylase activity variations. Out of four predicted outliers, leucine did not show a significant influence on amylase secretion (*P* = 0.76). While ME-models are the first generation of models with the ability to predict adapted amino acid synthesis profiles, numerous biological processes at the level of kinetics and regulation are not considered. Leucine is one of the amino acids with the lowest solubility in water, which significantly decreased the amount of amino acid that we could supplement in the samples, and thus reduces the driving force for its uptake in vivo. This physical limitation of leucine might have impacted the amylase activity increase.

## Discussion

Here, we constructed the *i*JT964-ME model of *Bacillus subtilis* and demonstrated its usefulness through gene essentiality predictions and three biologically and industrially relevant examples. The model contains 964 genes, 6282 reactions, and 4208 metabolites, and it captures the interdependence of genes, proteins, and metabolites, making it a significant improvement over the existing iYO844 M-model. *i*JT964-ME’s significantly expanded scope and more realistic expression framework led to a 40% increase in the precision of gene essentiality predictions, the removal of unrealistically high loop fluxes that plague unconstrained M-models, and an ability to model changes in amino acid metabolism and biomass functions.

ME-models can improve the prediction of flux distributions while overcoming inherent issues present in M-models. One of these issues that are most difficult to overcome is the prediction of metabolic loops with fluxes above biologically relevant ranges. In previous studies, loops have been addressed by coupling thermodynamic constraints^37^ and multi-step loopless algorithms^38^. In ME-models, both high and cyclic fluxes are penalized, as each unit of flux carries gene transcription, translation, and post-translational processing^20^. As a result, *i*JT964-ME eliminates previously present metabolic loops in the template M-model *i*YO844. This was shown for the simulated growth conditions and can be expected for any other simulation conditions. Moreover, ME-model architecture allows for a mechanistic interdependence between metabolic pathways. New reactions are essential due to new mechanisms in the network, and biomass precursor requirements adapt to different growth conditions. Thereupon, *i*JT964-ME was shown to improve gene essentiality predictions by 40% (MCC) as compared to *i*YO844.

Recent advances in transcriptomic analysis using machine learning tools have led to hypotheses about amino acid metabolism, which presented excellent questions for us to explore with our model. The study by Rychel et al.^25^ computed and characterized sets of co-regulated genes across a microarray dataset and identified signals with a lack of prior literature: (i) activation of tryptophan synthesis by ethanol stress and (ii) downregulation of arginine synthesis by salt shock. By simulating these conditions, we demonstrate that our model recapitulates these effects, without adding further constrains to the model, such as varying biomass reaction coefficients^12^ or condition-specific flux constraints^13^. With regard to (i), our model supports potential mechanisms involving increased amino acid synthesis for ethanol processing and efflux enzymes, and a potential role of folate degradation. For (ii), we observed decreased arginine synthesis as a result of overall downregulation for amino acid synthesis as expression flux shifts toward carbohydrate metabolism. It is worthy to note that in all cases, *i*YO844 could not capture any change in amino acid metabolism due to it being directly constrained by the fixed requirements in the biomass reaction.

In a final analysis, we assessed whether *i*JT964-ME could be employed to predict protein secretion at different growth conditions. For this, we reproduced two previous reports of amylase secretion at high^32^ and low^31^ growth rate conditions in silico. The model successfully predicted the secretion rate of amylase within the experimental reported range, though the secretion rate under low growth was overestimated. Overall, the dependence of growth rate on the required secretion rate of amylase was accurately captured. We performed a PCA to identify critical drivers of the metabolic change between these two conditions. As expected, the response to a higher expression of amylase was mixed with the global increase in metabolic activity. This showed the main drivers to be essential reactions related to biomass precursor synthesis, energy metabolism, and carbon source breakdown.

Thereupon, we isolated the response of amylase overproduction by simulating an increase in amylase secretion at a fixed growth rate. The new PCA unraveled key metabolic pathways directly related to amylase composition, with the biosynthetic pathways of the most abundant amino acids in its sequence having the highest weights in the principal components. However, the link between composition and weight in the principal component was not direct, as the amino acids with the highest weight were not always the most abundant. Out of the four predicted outliers with outstanding effect on amylase secretion, only one observation diverged from the simulations. Further, low-sensitivity amino acids were correctly predicted, with the notable case of cysteine, which was correctly predicted to negatively affect amylase secretion.

The iJT964-ME model represents a significant advancement in the metabolic modeling of *B. subtilis*. Its wide scope and ability to capture expression changes have improved gene essentiality predictions, shed light on recent hypotheses relating amino acid metabolism and stress, and explored the capacity to secrete industrially relevant proteins. This model can serve as the basis for unraveling further questions about metabolism and has the potential to be the foundation on which to optimize heterologous protein expression in this important model organism and cell factory.

## Methods

### Model reconstruction

Reconstruction was performed in Python 3.6, using the reconstruction packages cobrapy 0.5.4^22^ and COBRAme^20^. Models were solved using the package SOLVEme^39^. The *E. coli* reconstruction package ECOLIme^20^ was adapted with *B. subtilis* gene expression machinery, complexes, and translocation pathways. In brief, every reaction in a template core metabolic network (M-model) is coupled with the consumption of the enzyme that catalyzes it. Similarly, enzyme production pathways (transcription, translation, post-translational modification) are coupled with the corresponding catalyzing enzymes. The link of the reactions is performed with coupling coefficients, which represent the usage requirement of the catalyzing enzyme per unit flux of reaction.

The core metabolic network was taken from the available M-model *i*YO844, along with its gene-reaction rules (with updates presented in Supplementary Table 1). The used information is summarized and shown in Table 1.

### Flux prediction

Metabolic and gene expression flux distribution was predicted following the same protocol and software used in the reconstruction of the *E. coli* ME model *i*JL1678-ME^20^. Like an M-model, *i*JT964-ME is solved by finding a vector of flux rates, *v*, that maximizes biomass production while satisfying $S \ast v = 0$, where *S* is a matrix of dimensions $\left| M \right| \times \left| R \right|$ containing the stoichiometric coefficients of all metabolites in *M* in every reaction in *R*. The formulation of ME-models represents a nonlinear programming problem, which must be solved iteratively^40^. We solved flux distributions in *i*JT964-ME with SOLVEme^39^, which uses a binary search algorithm that looks for the maximum possible growth rate that is feasible. In each iteration, a growth rate was assumed and substituted in all symbolic expressions to yield a linear programming problem (LP). Then, the QuadMINOS^40^ solver was called to solve the LP and assess feasibility in quad-precision.

### Carbon substrate analysis

As a way of checking the sanity of the metabolic network, we reproduced the carbon substrate analysis performed by Oh et al.^7^ with the template M-model *i*YO844. Only those carbon substrates that were already present in the model were included in this analysis, which leaves 88 carbon substrates. Detailed results are shown in Supplementary Data 1.

### Gene essentiality analysis

Single gene knockouts were modeled by closing their respective translation reactions. Genes were deemed essential when the single knockouts resulted in a growth rate of zero. Results were validated with a list of essential genes reported by Juhas et al.^41^ for *B. subtilis* and EcoCyc^42^ for *E. coli*. Gene functions were assigned as annotated in the subsystem of the reaction they catalyze. Metabolic subsystem annotation was taken from *i*YO844, and gene expression subsystems were assigned according to the catalyzed reaction types (as shown in the reaction breakdown in Fig. 1b).

Scores used to assess the performance of the gene essentiality predictions are True Positive Rate (TPR), True Negative Rate (TNR), False Discovery Rate (FDR), Matthews Correlation Coefficient (MCC), Precision, and Coverage. The definitions were as follows:

1 $${{{\mathrm{TPR}}}} = \frac{{{{{\mathrm{TP}}}}}}{{{{{\mathrm{TP}}}} + {{{\mathrm{FN}}}}}}$$

2 $${{{\mathrm{TNR}}}} = \frac{{{{{\mathrm{TN}}}}}}{{{{{\mathrm{TN}}}} + {{{\mathrm{FP}}}}}}$$

3 $${{{\mathrm{FDR}}}} = \frac{{{{{\mathrm{FP}}}}}}{{{{{\mathrm{FP}}}} + {{{\mathrm{TP}}}}}}$$

4 $${{{\mathrm{MCC}}}} = \frac{{\left( {{{{\mathrm{TP}}}} \ast {{{\mathrm{TN}}}} - {{{\mathrm{FP}}}} \ast {{{\mathrm{FN}}}}} \right)}}{{\left( {{{{\mathrm{TP}}}} + {{{\mathrm{FP}}}}} \right) \ast \left( {{{{\mathrm{TP}}}} + {{{\mathrm{FN}}}}} \right) \ast \left( {{{{\mathrm{TN}}}} + {{{\mathrm{FP}}}}} \right) \ast \left( {{{{\mathrm{TN}}}} + {{{\mathrm{FN}}}}} \right)}}$$

5 $${{{\mathrm{Precision}}}} = \frac{{{{{\mathrm{TP}}}}}}{{{{{\mathrm{TP}}}} + {{{\mathrm{FP}}}}}}$$

6 $${{{\mathrm{Coverage}}}} = \frac{{{{{\mathrm{Number}}}}\,{{{\mathrm{of}}}}\,{{{\mathrm{genes}}}}\,{{{\mathrm{in}}}}\,{{{\mathrm{model}}}}}}{{{{{\mathrm{Total}}}}\,{{{\mathrm{number}}}}\,{{{\mathrm{of}}}}\,{{{\mathrm{genes}}}}\,{{{\mathrm{in}}}}\,{{{\mathrm{database}}}}}}$$

### Modeling ethanol stress

Ethanol is a small polar molecule that can readily diffuse through the cell membrane. Therefore, ethanol uptake was modeled with no enzymatic coupling. The exchange reaction of ethanol was opened with lower and upper bounds equal to a defined uptake rate (see uptake rates in Fig. 2a), while all other exchange constraints were left unchanged as defined in ref. ^7^. Transcription and translation levels were defined as a ratio of the flux at a specific condition to the maximal flux in the whole dataset to normalize and highlight trends.

### Modeling salt stress

As opposed to ethanol, salt is transported through the membrane by a series of complexes. Transcriptomics data under salt stress showed that salt importers were downregulated, implying that an increased flux of salt occurs without a higher expression of transporters. Therefore, salt stress was modeled by an artificial uptake of salt uncoupled to any transporter so that higher uptakes did not falsely trigger importer expression in the model. Secretion complexes were left unchanged and coupled to salt secretion pathways. As in the simulation of ethanol stress, sodium uptake bound was defined as the different uptake rates, while all other constraints were left unchanged as defined in *i*YO844^7^.

### Modeling and validating amylase secretion rates

In the model, amyE is transcribed (transcription_TU8J2_1134_from_BSU25200-MONOMER), translated (translation_BSU25200), secreted through the sec pathway (translocation_BSU03040), and finally used in the hydrolysis of extracellular starch (AAMYL_1_FWD_BSU03040-MONOMER). Secretion rates, in units of mmol gDW^−1^ h^−1^, correspond to the flux through translocation_BSU03040. Random sampling of the solution space was performed from 90 to 100% of the optimal growth rate at the simulation conditions, in order to generate a robust distribution of biologically relevant fluxes^12,34^. First, the model was solved at low and high-amylase expression conditions to obtain lower and upper bounds of all exchange reactions in the model, thus yielding ranges of exchange reactions that ensure model feasibility (see “Flux prediction”). Then, exchange reactions were constrained with random values within the calculated bounds in every sampling iteration. This solution space was sampled 100 times.

Validation of secretion rates was performed in data collected from two previously reported experimental datasets, at a high^32^ and low^31^ growth rate. In both studies, the authors reported time-course biomass, X (g L^−1^), and amylase activity, A (mU mL^−1^). Amylase activity required conversion to mass concentration for direct comparison with model predictions. Thus, we converted activity to amylase concentration, C (mg mL^−1^), using a typical range for *B. subtilis*’ amylase specific activity^43^ of 153.7 (minimum) to 245 (maximum) U mg^−1^. Then, growth and amylase secretion rates were obtained using a linearized model.

Equations (7) and (8) are the mass balance equations for biomass (*X*) and amylase (C) in batch culture, where μ is the growth rate and *v* is the amylase secretion rate. Integration for ${{{\mathrm{X}}}}\left( {{{\mathrm{t}}}} \right)$ in Eq. (7) yields the linearized model $\log \left( {{{\mathrm{X}}}} \right) = \mu {{{\mathrm{t}}}} + {{{\mathrm{k}}}}$. Growth rate was then estimated by fitting the datapoints during exponential growth to the linearized model. The Pearson correlation coefficients of the linear regressions for the datasets at high^32^ and low^31^ growth rate conditions were 0.81 and 0.98, respectively. For the amylase secretion rate (ν), integration for $\left. {{{{\mathrm{C}}}}\left( {{{\mathrm{t}}}} \right)} \right|_{t_0}^{t_f}$ and solving for ν in Eq. (8) yields Eq. (9).

7 $$\frac{{{{{\mathrm{dX}}}}}}{{{{{\mathrm{dt}}}}}} = \mu {{{\mathrm{X}}}}$$

8 $$\frac{{{{{\mathrm{dC}}}}}}{{{{{\mathrm{dt}}}}}} = \nu {{{\mathrm{X}}}}$$

9 $$\nu = \frac{{{\mathrm{C}}_{\mathrm{f}} - {\mathrm{C}}_0}}{{\mathop {\int }\nolimits_{{\mathrm{t}}_0}^{{\mathrm{t}}_{\mathrm{f}}} {{{\mathrm{Xdt}}}}}}$$

### Modeling the overexpression of amylase

Amylase overexpression was performed by setting the growth rate constant at the minimum rate exhibited in the distribution of samples at a high growth ($0.17\,{{{\mathrm{h}}}}^{ - 1}$). Then, amylase secretion rate was randomly forced within the range from the base requirement at $0.17\,{{{\mathrm{h}}}}^{ - 1}$ until the requirement the model would predict for $0.195\,{{{\mathrm{h}}}}^{ - 1}$. This solution space was sampled 100 times.

### Principal component analysis to identify critical reactions

Principal Component Analysis (PCA) is a resourceful unsupervised machine learning method that identifies key underlying variables that drive the variance in the samples. We used PCA to identify key reactions that explain the variance between high and low growth, as well as within the samples at different levels of amylase overexpression. The flux data were pre-processed by calculating z-scores and then fed to the scikit-learn method sklearn.decomposition.PCA.

### Growth of *B. subtilis* and amylase activity

*B. subtilis* strain 168 was struck out on LB agar plate overnight. One colony was inoculated in LB medium for 16 h at 37 °C, pelleted and resuspended in M9 medium. For amylase production testing, the cell resuspension was inoculated into M9, M9 + 0.2% starch (through 0.22uM filter), and M9 + 0.2% starch + amino acid in a 96-well plate at the starting inoculum of OD600 = 0.07 and final volume of 200ul. All amino acid concentrations were at 20 mM when possible, based on solubility data provided by the manufacturer (Millipore Sigma). Exceptions are Asp (3.4 mM), Glu (5.8 mM), Ile (14 mM), Leu (3.8 mm), Phe (16.3 mM), Trp (6.6 mM), Try (0.3 mM). All growth conditions were set up in triplicates. After 24 h at 37 °C, amylase activity and production were determined using the Sigma-Aldrich® Amylase Activity Assay Kit (MAK009). The protocol was followed exactly as suggested by the assay kit manufacturer. OD_405_ was measured at 24 h, and the value of the starch-only sample was subtracted from the value of each amino acid- supplemented sample. Amylase activity was reported as nmole/min/mL (milliunits), considering that one unit of amylase is the amount of amylase that cleaves ethylidene-pNP-G7 to generate 1.0 mmole of p-nitrophenol per minute at 25 °C.

### Sampling of cysteine synthesis rates

Sampling was performed in two different amylase secretion conditions, corresponding to amylase secretion rates calculated from ref. ^31^ (low amylase secretion) and ref. ^32^ (high-amylase secretion). Accordingly, amylase secretion rates were centered around 1.36e-8 and 4.4e-7 mmol Amylase gDW^−1^ h^−1^, allowing for a 10% variation. Sampling was performed with 100 points, and outliers were removed with 95% confidence. Significance in the change of cysteine synthesis rate distributions across both conditions was assessed with a two-tailed *t* test. The sensitivity of cysteine synthesis to amylase expression, in units of ${{{\mathrm{mmol}}}}\,{{{\mathrm{Cys}}}} \ast {{{\mathrm{mmol}}}}\,{{{\mathrm{Amylase}}}}^{ - 1}$, was calculated as shown in Eq. (10). In this equation, $\bar x_{high}$ and $\bar x_{low}$ represent the means of cysteine secretion rates in the distribution of fluxes at the high and low amylase expression conditions, respectively. Similarly, $r_{high}$ and $r_{low}$ represent the rates of amylase expression at the high and low amylase expression conditions, respectively.

10 $$sensitivity = \frac{{change\,in\,cysteine\,synthesis}}{{change\,in\,amylase\,expression}} = \frac{{\bar x_{high} - \bar x_{low}}}{{r_{high} - r_{low}}}$$

## Supplementary information

Supplementary Information

Flux distributions of the M- and ME-models under different substrates

Sensitivity, amino acid composition and observed effect in amylase activity of amino acids.

Raw measurements of amylase activity with amino acid supplementation

Sampling flux distributions at low and high amylase secretion rates.

Raw measurements of biomass concentration with amino acid supplementation

## Data Availability

All relevant data are contained in this document, the supplementary files, and the repository available at www.github.com/jdtibochab/bacillusme. Metabolic models are provided following the standard protocols for computational analysis^44^.

## Code availability

The repository including the code related to this study is available at www.github.com/jdtibochab/bacillusme.
