# Supplementary material for: Predicting stress response and improved protein overproduction in Bacillus subtilis
Source: NPJ Syst Biol Appl. 2022 Dec 27;8:50. doi: 10.1038/s41540-022-00259-0 (PMC9794813; doi:10.1038/s41540-022-00259-0)
Supplement: Supplementary file 1 — Supplementary Information [file 41540_2022_259_MOESM1_ESM.pdf]

Supplementary Information

Predicting stress response and improved protein overproduction in *Bacillus subtilis*

Juan D. Tibocha-Bonilla[1], Cristal Zuñiga[2,3], Asama Lekbua[4], Colton Lloyd[5], Kevin Rychel[5], Katie Short[4], Karsten Zengler[2,5,6]*

This file contains:

Supplementary Figure 1

Supplementary Figure 2

Supplementary Figure 3

Supplementary Figure 4

Supplementary Table 1

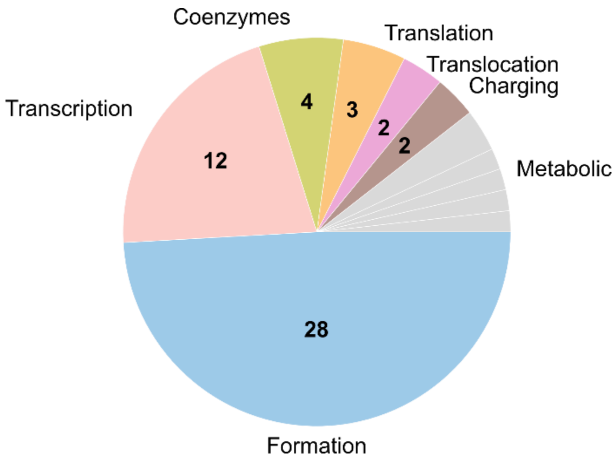

Supplementary Figure 1. Genes added in the ME-model reconstruction that are predicted to be essential but have been observed as non-essential (false positives). Gene expression machinery accounts for the largest portion of false positives in our essentiality predictions, mainly complex formation, transcription, translation, translocation, and tRNA charging. Moreover, cofactor synthesis for enzyme activation appears as third largest contributor.

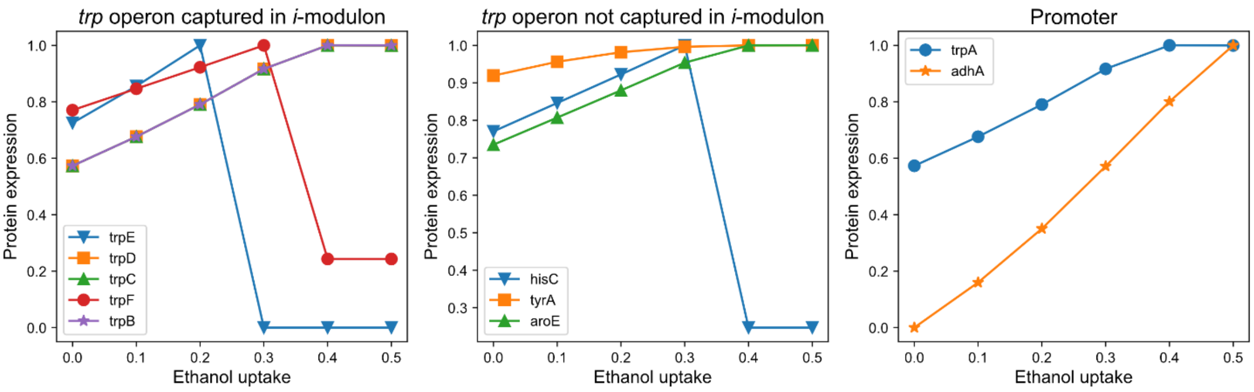

Supplementary Figure 2. Translation rates of genes in the *trp* operon at varying ethanol uptake rates. Genes are presented separated considering whether they were called in the *trp i*-modulon in the study by Rychel et al.[26]

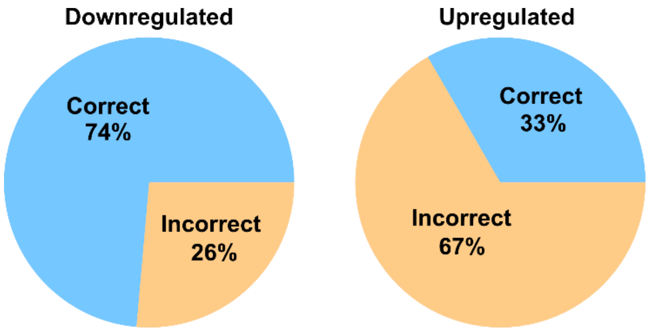

Supplementary Figure 3. Breakdown of correctly and incorrectly predicted differential expression at the genome scale under salt stress. Downregulated genes are well predicted with a 74% accuracy, while upregulated genes contain numerous false positives coming from storage compound biosynthesis.

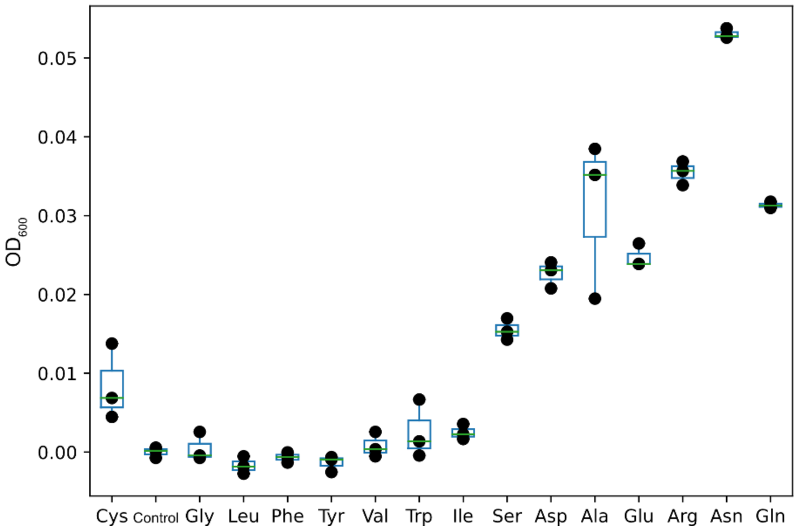

Supplementary Figure 4. Effect of amino acid supplementation on final biomass concentrations (OD_{600}). Growth rate is directly affected by amino acid supplementation. Nutritional value of amino acids can be one of the main contributors to supplementation effect on amylase secretion and growth, although it does not explain the effect of cysteine, aspartate, glutamate, and glutamine.

Supplementary Table 1. Updates of transporters and gene-protein-reaction associations in *i*YO844.

Reaction ID	Corrected GPR rule
**ACTD2**	BSU08060 and BSU08070
**ACt2r**	BSU38240
**AIRC1**	BSU06420 or BSU06430
**ANS**	BSU00750 or BSU22680
**ASPt2r**	BSU10220
**CBLtex**	BSU33170
**F6Pt6_2**	BSU12010 or BSU14400
**GLCpts**	BSU13890 or (BSU38570 and BSU38580 and BSU38590)
**HCO3E**	BSU30690
**Kt3r**	BSU31610 and BSU31660 and BSU31600 and BSU3162…
**LYSLG_BS**	-
**MAN6Pt6**	BSU10520
**NAt3_1**	BSU31600 and BSU31610 and BSU31620 and BSU3163…
**PGL**	BSU13010
**PNTOt2**	-
**PRFGS_1**	BSU06480 or BSU06470
**PYRt2**	BSU28900 and BSU28910
**RIBFLVt2**	BSU23050
**RNDR1**	BSU17380 and BSU17390
**RNDR2**	BSU17380 and BSU17390
**RNDR3**	BSU17380 and BSU17390
**RNDR4**	BSU17380 and BSU17390
